# The effects of invasive pests and pathogens on strategies for forest diversification

**DOI:** 10.1016/j.ecolmodel.2017.02.003

**Published:** 2017-04-24

**Authors:** Morag F. Macpherson, Adam Kleczkowski, John R. Healey, Christopher P. Quine, Nick Hanley

**Affiliations:** a Computing Science and Mathematics, School of Natural Sciences, University of Stirling, Cottrell Building, Stirling FK9 4LA, UK; b School of Environment, Natural Resources and Geography, College of Natural Sciences, Bangor University, Bangor, Gwynedd LL57 2UW, UK; c Forest Research, Northern Research Station, Roslin, Midlothian EH25 9SY UK; d School of Geography & Geosciences, Irvine Building, University of St Andrews, North Street, St Andrews, Fife KY16 9AL, UK

**Keywords:** Bioeconomic modelling, Forest management, Natural resource management, Tree pests and pathogens, Tree species diversification

## Abstract

Diversification of the tree species composition of production forests is a frequently advocated strategy to increase resilience to pests and pathogens; however, there is a lack of a general framework to analyse the impact of economic and biological conditions on the optimal planting strategy in the presence of tree disease. To meet this need we use a novel bioeconomic model to quantitatively assess the effect of tree disease on the optimal planting proportion of two tree species. We find that diversifying the species composition can reduce the economic loss from disease even when the benefit from the resistant species is small. However, this key result is sensitive to a pathogen's characteristics (probability of arrival, time of arrival, rate of spread of infection) and the losses (damage of the disease to the susceptible species and reduced benefit of planting the resistant species). This study provides an exemplar framework which can be used to help understand the effect of a pathogen on forest management strategies.

## Highlights

• Novel bioeconomic model assesses effect of tree disease on tree species mixtures.
• Risk and damage of disease alters the optimal planting proportion of two species.
• Diversifying reduces loss from disease even if resistant species benefit is small.
• Optimal planting proportion sensitive to disease characteristics and economic loss.

## 1 Introduction

Tree pest and pathogen outbreaks can have negative economic and environmental impacts, especially when large areas of forest are affected (Pimentel et al., 2005, Ayres and Lombardero, 2000). Once a pest or pathogen has established there are relatively few treatments that help diseased trees to recover, therefore any reactive strategy tends to focus on controlling the outbreak (often this is preventing or reducing the spread to other forest areas). On the other hand, anticipatory (proactive) strategies have been proposed to reduce the initial susceptibility of forests to an outbreak, and/or to reduce the impact of disease on the trees once a pest or pathogen has arrived (Quine et al., 2017, Jactel et al., 2005, Jactel et al., 2009, Wainhouse, 2004). In this study, a mathematical model is used to examine one such strategy, and in particular to address the question: how does the arrival of a pathogen and occurrence of disease affect the optimal planting strategy with respect to including a second tree species in a mixture?

The literature examining the effect of diversification of the tree species composition of forests on timber and non-timber outputs is ever expanding; however, the range of ecological impacts are difficult to disentangle and explicitly define (Jactel et al., 2009). The type of forest and the objective(s) of the forest owner or social planner will influence the economic and ecological outcomes of diversifying. In this paper, the focus is narrowed by considering a plantation where the manager is interested in the productivity of timber only. Plantation forests are commercially important since they contribute a large proportion of timber to the world markets. They often consist of a single species monoculture chosen for growth or other properties, but are potentially vulnerable to a pest or pathogen of that tree species. For example, over the last century eucalyptus (species of *Eucalyptus* and *Corymbia*) has been grown in non-native plantations in large areas of the southern hemisphere. Their fast growth rate, and separation from their natural enemies has made them an economically important species in South America, South Africa, and more recently South and East Asia (Wingfield et al., 2008). However, the increase in arrival of pests and pathogens, such as cryphonectria canker caused by the fungus *Cryphonectria cubensis* (Wingfield, 2003), is beginning to have a negative affect (Wingfield et al., 2008). Another example is *Ips typographus* that has been shown to have a greater effect on stands with higher proportions of spruce trees (Wermelinger, 2004). Due to the high proportion of *Picea sitchensis* monocultures in the UK, a contingency plan (Forestry Commission, 2015) has been created in case the beetle is found.

With world trade generating a high level of new species invasions (Brasier, 2008), strategies to reduce the impact of pests and pathogens on plantations are of great importance. Species diversification is one such strategy. The main argument for diversifying the tree species composition of production forests is the “insurance hypothesis” since, at the forest level, planting more than one species spreads the risk (Loreau et al., 2001, Pautasso et al., 2005). This means that the initial susceptibility and/or the impact is reduced if a pest or pathogen does arrive, particularly as many are species- or genus-specific in their impact. Modelling in Sweden has shown that there is a reduction in the risk of damage from *Heterobasidion annosum* when *Picea* stands are mixed with *Pinus* (Thor et al., 2005), moreover transmission rates of *Armillaria* spp. were found to reduce with increased tree diversity by Gerlach et al. (1997). Haas et al. (2011) used field data and Bayesian hierarchical models to show that sites with higher species diversity have a reduced disease risk of *Phytophthora ramorum* in California, and the experiments of Hantsch et al. (2014) showed that local tree diversity can decrease the level of fungal pathogen infestations of *Tilia cordata* and *Quercus petraea*. More recently, Guyot et al. (2016) sampled a network of forest plots spanning several countries, and showed a positive relationship between tree species richness and resistance to insect pests. They argued that these “findings confirm the greater potential of mixed forests to face future biotic disturbances in a changing world” (Guyot et al., 2016).

Bioeconomic models can be a useful tool to examine the effect of pests and pathogens on forest management strategies such as species diversification (we provide a short literature review of this research area in Section 2). In this paper, we create a bioeconomic model that finds the optimal planting strategy for two tree species. It is assumed that a forest manager has the option of planting two tree species (species A or species B or both), over a fixed rotation period (note: we consider the effects on optimal rotation for a single species in Macpherson et al., 2016, Macpherson et al., 2017). We assume that in the absence of a pathogen threat, the commercially preferred species is species A. However, species A is susceptible to a new pathogen that will lower the timber benefit; whereas species B is resistant. The optimal planting strategy, more specifically how much of each species to plant, is the strategy that minimises the expected economic loss.

The mathematical framework for this optimisation problem consists of an objective function that calculates the expected present value loss of planting both species, when compared with the ‘ideal situation’ of a monoculture of trees of species A remaining un-infected. The potential loss due to planting trees of species A will depend on a number of factors: the probability of arrival of the pathogen and occurrence of disease; when the pathogen arrives within the rotation; how fast the pathogen spreads throughout the forest; and the effect of the disease on the timber benefit (through increased harvesting costs, or reduced growth or reduced quality of the timber). Thus, the objective function depends on the area of infected trees (of species A) at the end of the rotation, which is described by a Susceptible-Infected epidemiological compartmental model.

How fast the pathogen spreads throughout the forest will largely depend on the contact rate (for example, of spores) with a tree, the probability that contact is with a tree of species A, and the probability that the tree is susceptible to disease. This formulation will depend on the spatial arrangement of the trees within the forest, since the probability that contact is made with a tree of species A, will likely be different if species A is planted in a monoculture block, or in an intimate mixture with species B. Whilst we do not explicitly define space in the model, we demonstrate how the pathogen transmission term is constructed for both a monoculture and an intimately mixed forest. Exploring both these cases is important, since the majority of the existing evidence reported above shows a positive effect of tree species diversity (on reducing the effect of disease) when the species in the forest plots are intimately mixed.

The two research questions that this paper addresses are: (1) what is the optimal planting strategy when species A returns a higher timber benefit than species B, but species A is susceptible to a new disease, whereas species B is not, and (2) how do different bioeconomic conditions alter the optimal planting strategy? Examining these questions for a range of bioeconomic parameter sets facilitates a better understanding of the qualitative effects that pathogen characteristics can have on the optimal planting strategy, since our model is not based on a specific host–pathogen system.

The layout of the paper is as follows. A short literature review on using bioeconomic models to analyse the effect of pests and pathogens on forest management strategies is given in Section 2. The economic and epidemiological components of the model are derived in Section 3, and the results are given in Section 4. A discussion in Section 5 is followed by a brief conclusion of the key results found in this paper in Section 6.

## 2 Bioeconomic modelling of the effect of pests and pathogens on forest management strategies

Changing forest management strategies in response to a pest or pathogen threat often has major economic consequences (Wainhouse, 2004). For example, there will likely be a cost of changing the strategy but, if successful, after a pest or pathogen arrives, the forest output (timber and/or non-timber) may be maintained at a higher level, and thus there will be a benefit (compared with ‘doing nothing different’). The decision maker therefore has to weigh-up the costs and benefits of changing the strategy, with the risk of the pest or pathogens arriving, and their predicted effect on the forest.

Mathematical modelling has been used to examine these effects ‘in silico’. Models can help to analyse and compare the effect of a pest or pathogen on the relative success of alternative management strategies under different economic and biological conditions. This section highlights some of the bioeconomic models that have been developed to analyse: forest management strategies in the presence of a pest or pathogen; invasion–specific management strategies such as surveillance or control; and the effect of mixed species composition in the presence of other abiotic and biotic risks. (Note that the difference between the first and second cases is that the first assumes that a *change* in a management strategy occurs (i.e. these strategies occur when there is no risk of an incursion), whereas ‘invasion specific’ strategies are deployed specifically to target management of a pest or pathogen risk.)

There are many forest management strategies whose success may be affected by a pest or pathogen incursion. Jactel et al. (2009) reviewed the effect of a range of forestry practices on biotic and abiotic hazards. Strategies shown to affect the likelihood of an outbreak, and susceptibility of forests to pathogens and pests, included thinning and pruning, tree species composition and density of planting. Using knowledge from practitioners and experts Quine et al. (in preparation) recommended 33 strategies as potentially relevant to combat *Dothistroma septosporum*, in just one country, the UK. Bioeconomic models can be used to explore the effect that disease may have on this multiplicity of alternative strategies, which would be very time consuming to individually test empirically. However, despite their benefits, bioeconomic models are still underutilised in examining how the optimality of strategies changes in the presence of disease.

An example of the insight bioeconomic models can give has been demonstrated for *H. annosum*, an economically important pathogen of conifers. *H. annosum* is widespread in Europe and spreads through spores which colonise freshly cut conifer stumps, causing timber deterioration and thus a reduction in its commercial value. Several models have been used to simulate the spread of the pathogen, at a tree and forest level, and the subsequent timber decay (Seifert, 2007, Pukkala et al., 2005). Moreover, bioeconomic models (which combine both pathogen dynamics and economics) have been used to examine the effect of management strategies, such as thinning and chemical stump treatment, on the reduction of the pathogen spread and economic damage (Wang et al., 2015, Thor et al., 2006, Möykkynen and Miina, 2002).

Bioeconomic models have also been used to assess the effect of a pathogen on the optimal rotation length of forests (Macpherson et al., 2016, Macpherson et al., 2017). The authors adapted a classical Faustmann model to include the rate of pathogen spread (through a Susceptible-Infected epidemiological model). This optimal control framework showed that the optimal rotation length (the forest age at which net present value of the forest is maximised) of a plantation forest is generally shortened when the damage from disease reduces the timber benefit (Macpherson et al., 2016). When a forest manager considers both the timber and non-timber benefits, and the damage from disease reduces the timber benefit only, the optimal rotation length increases; when the damage reduces both the timber and the non-timber benefits, the optimal rotation length is reduced (Macpherson et al., 2017).

Other bioeconomic models that address tree disease management strategies which aim to reduce the impact of an invasion, focus on questions about optimal surveillance (Epanchin-Niell et al., 2014) and control (Thompson et al., 2016, Mbah and Gilligan, 2010, Mbah and Gilligan, 2011, Sims et al., 2010, Thor et al., 2006). Epanchin-Niell et al. (2014) created a mechanistic bioeconomic model to examine the cost-efficiency of a trap-based pest surveillance program for multiple, simultaneous, novel invasions at a landscape scale. In their model, multiple pests arrive, spread and cause damages to urban and plantation forests, but upon detection eradication can be attempted at a cost (dependent on the area of the invasive species population). Earlier detection can lead to a greater chance of eradication, and reduction in the future damages and losses. The authors use a case study of wood borer and bark beetles in New Zealand to parameterise their model, and found the optimal surveillance program, which minimised the total net present value of expected future costs (surveillance, invasion damage, and control costs), required very high investment in surveillance (about 10,000 traps in each year of the 30-year surveillance program). This strategy reduced the costs by 39% compared with no surveillance (Epanchin-Niell et al., 2014); moreover in general they found that the cost, even at a low level of surveillance, was offset by the economic benefits of surveillance.

In Sims et al. (2010), a bioeconomic model is used to examine passive, localised and centralised timber harvesting strategies to maximise a household utility function (which included both produced goods, like timber, and the quality of a *Pinus contorta* forest, such as recreation, amenity values, and ecosystem services) with a *Dendroctonus ponderosae* outbreak. The baseline strategy of passive management involved no control or harvesting, whereas the localised and centralised strategies involved the forest manager harvesting adult and salvage (dead) trees dependent on household preferences, the stock of trees and the *D. ponderosae* population. The difference between these strategies is that localised management optimally treats the outbreak as exogenous (the harvest decisions are made in response to an outbreak and take future outbreaks as given), whereas centralised management optimally recognises the endogenous nature of *D. ponderosae* (the harvest decisions are made considering future outbreaks and future tree mortality). The authors found that centralised forest management substantially reduced the size of the outbreaks and risk of future outbreaks, when compared with passive and localized management (which actually increased the risk and severity of epidemics).

Models have also been used to assess the general risk of a catastrophic event on mixed species forests (Griess and Knoke, 2013, Neuner et al., 2013, Roessiger et al., 2013, Knoke et al., 2005, Knoke and Seifert, 2008). The majority of these papers use portfolio theory (Markowitz, 1952) to establish the expected financial return and risk of investment in a forest. For example, Knoke et al. (2005) evaluated mixed *vs.* single species management of *Picea abies* and *Fagus sylvatica*. The expected financial return (net present value of all future net revenue flows) and the risk of the investment were calculated by using Monte Carlo simulations. Planting a mixed-species forest (where the species were planted in separate blocks) reduced the profitability due to the lower value of *F. sylvatica* compared with *P. abies*. However, increasing the risk of planting *P. abies* (through an increased risk of the occurrence of a natural hazard), reduced the return from *P. abies*, made planting a mixture more profitable and reduced the overall risk of the portfolio (Knoke et al., 2005). In a follow-up study, Knoke and Seifert (2008) used a bioeconomic model to examine the financial return and risk of two different forest types of the same two species – a pure *F. sylvatica* forest and a mixture (planted in smaller rectangular blocks). The authors used data from existing studies on forest productivity, timber quality and resistance to the hazard (a polynomial survival probability function, based on storm damage data) to inform the model. Again, Monte Carlo simulations, under site conditions and risks typical of southern Germany, were used to simulate the financial risk and return for varying proportions of species in the mixture. The main results showed that a mixture decreased the financial risk and increased the return when all the tested ecological factors were included (Knoke and Seifert, 2008).

The difference between a pest or pathogen outbreak and other abiotic risks, such as fire or storms, can be significant (for example, the time scale over which the event occurs, the symptoms and whether it leaves salvageable timber). We therefore argue that a separate study is required to examine the effect of a pathogen on the success of forest management strategies differing in tree species diversification. Moreover, previous studies concentrate on specific host–pathogen systems, which can be necessary when addressing strategies to combat single pathogen species (Thompson et al., 2016, Mbah and Gilligan, 2011, Sims et al., 2010, Thor et al., 2006). However, much benefit can be gained by developing and analysing general models that highlight the interaction of a general pest or pathogen with the management strategy, and allow the sensitivity to biological and economic parameters to be investigated. This also has the advantage of identifying which parameters are important when considering a specific host–pathogen system, and so help prioritise data gathering.

## 3 Model framework

First, we list some terminology used throughout this paper. The total area of the forest managed is referred to as the ‘plot’. A ‘monoculture’ refers to a planting strategy where only one tree species is planted in the plot, whilst a ‘mixture’ refers to the case when two species are planted intimately, with no spatial aggregation of the trees of each species throughout the plot. In this section, the model is formulated in two parts. The first derives the minimisation problem for two scenarios (Section 3.1), and the second creates a Susceptible-Infected (SI) compartmental model (Section 3.2). All parameter definitions and values can be found in Table 1.

### 3.1 Economic model

The area of the plot is fixed at *L* hectares, and the parameter *δ* controls the fraction of species B that is planted, where *δ* ∈ [0, 1]. Therefore, the area occupied by trees of species A is *L*_*A*_(*δ*) = (1 − *δ*)*L*, and the area occupied by trees of species B is *L*_*B*_(*δ*) = *δL*. We assume that the cost of establishment is the same for both species. Similarly the silvicultural practices, which are implemented throughout the rotation, are the same and obtain the same results. The difference between the two species is realised through the timber benefit obtained at the end of the rotation (the rotation length is fixed as *T* = 40 years for both species and we do not allow early felling).

The net benefit of species A without disease is a product of the price of timber per cubic metre, *p*, the timber volume per unit area at the end of the rotation, *f*(*T*), and the area of the plot, *L*. The probability that a pathogen will arrive at the plot during the rotation is *P* where *P* ∈ [0, 1] (for the purpose of this paper, the forest manager is assumed to have full knowledge of this probability and to be risk neutral). If the pathogen arrives and disease occurs, then the timber benefit from trees of species A is reduced through either lower timber quality (for example, due to staining or rot causing loss of mechanical integrity), slower timber growth, greater costs of harvesting, or through lower price due to local market saturation with that species. To include this in the model, the function ${\tilde{L}}_{A}(T,\delta )$ is used to represent the effective area of the forest occupied by species A at the end of the rotation when disease is present (explained in detail in Section 3.2). If species B is planted, a reduced timber benefit occurs through a slower growth rate or reduced timber value (when compared with the net benefit from uninfected trees of species A); and a factor *R*_*P*_ is used to scale the value of timber from species B (relative to timber from uninfected trees of species A) where *R*_*P*_ ∈ [0, 1]. The optimal planting strategy is the strategy which minimises the expected loss in timber benefit when compared with the timber benefit of species A without disease. We now explain how the minimisation problem is set-up dependent on whether the forest manager is planting a monoculture (Section 3.1.1), or a mixture (Section 3.1.2).

#### 3.1.1 Planting a single species monoculture

First assume that the forest manager can only plant a monoculture of species *A* (*δ* = 0 giving *L*_*A*_(0) = *L*) *or* species *B* (*δ* = 1 giving *L*_*B*_(1) = *L*). We define *s* to be the strategy variable where *s* ∈ {*A*, *B*}. For strategy *s* = *A*, without disease the net benefit is *pf*(*T*)*L*, and with disease the net benefit is $\mathrm{pf}(T){\tilde{L}}_{A}(T,0)$. This gives the expected net benefit of $\left(1-P)\mathrm{pf}(T)L+\mathrm{Ppf}(T){\tilde{L}}_{A}(T,0\right)$. For strategy *s* = *B*, disease has no impact and the net benefit is *R*_*P*_*p f*(*T*)*L*. To find the optimal planting strategy, we first define an intermediate expected objective function which describes the expected net present *loss* of both strategies,

(1) $$E[\hat{J}(s)]=\left\{\begin{array}{cc} P\left(\mathrm{pf}(T)L-\mathrm{p f}(T){\tilde{L}}_{A}(T,0)\right)e^{-\mathrm{rT}}, & \mathrm{if}\;s=A\\ \left(\mathrm{pf}(T)L-R_{P}\mathrm{p f}(T)L\right)e^{-\mathrm{rT}}, & \mathrm{if}\;s=B, \end{array}\right)$$

where we discount the future benefit, with rate *r*. The minimisation problem is specified as

(2) $$\min_{s\in \{A,B\}}E[\hat{J}(s)].$$

Factorising *p f*(*T*)*e*^−*rT*^ from Eq. (1) (since it is independent of the choice of *s*), the minimisation problem is equaivalent to minimising the expected objective function

(3) $$E[J(s)]=\left\{\begin{array}{cc} P(L-{\tilde{L}}_{A}(T,0)), & \mathrm{if}\;s=A\\ L(1-R_{P}), & \mathrm{if}\;s=B. \end{array}\right)$$

Thus

(4) $$\min_{s\in \{A,B\}}E[\hat{J}(s)]\Leftrightarrow \min_{s\in \{A,B\}}E[J(s)],$$

and the optimal strategy s^*^ is given by

(5) $$s^{*}=\arg\min_{s\in \{A,B\}}E[J(s)].$$

#### 3.1.2 Planting a two species mixture

Now assume that a mixture of two species can be planted, and the optimal strategy is determined by the value of *δ* which minimises the expected loss from planting a mixture (compared with planting only species A and the trees remaining uninfected). The expected net benefit from the area of trees of species A, given a probability *P* of a pathogen arriving, is $\mathrm{P p}f(T){\tilde{L}}_{A}(T,\delta )$, and the net benefit from the area of trees of species B is *R*_*P*_*p f*(*T*)*L*_*B*_(*δ*). Therefore, the expected net present loss is given by an intermediate expected objective function

(6) $$E[\hat{H}(\delta )]=\mathrm{P p}f(T)\left(L-{\tilde{L}}_{A}(T,\delta )-R_{P}L_{B}(\delta )\right)e^{-\mathrm{rT}}+(1-P)\mathrm{p f}(T)\left(L-L_{A}(\delta )-R_{P}L_{B}(\delta )\right)e^{-\mathrm{rT}},$$

giving the minimisation problem

(7) $$\min_{\delta \in [0,1]}E[\hat{H}(\delta )].$$

As before, factorising *p f*(*T*)*e*^−*rT*^ from Eq. (6), the minimisation problem is equaivalent to minimising the expected objective function

(8) $$E[H(\delta )]=P\left(L_{A}(\delta )-{\tilde{L}}_{A}(T,\delta )\right)+L_{B}(\delta )\left(1-R_{P}\right).$$

Thus

(9) $$\min_{\delta \in [0,1]}E[\hat{H}(\delta )]\Leftrightarrow \min_{\delta \in [0,1]}E[H(\delta )],$$

and the optimal strategy δ^*^ is given by

(10) $$\delta^{*}=\arg\min_{\delta \in [0,1]}E[H(\delta )].$$

#### 3.1.3 Timber volume

The net benefit at the end of the rotation is dependent on the volume of timber per unit area, which in this paper is *f*(*T*) = 579.9 m^3^ ha^−1^ when *T* = 40. This value is taken from the Forest Yield model, which has been developed by the UK government agency Forest Research, and is used to estimate the average timber volume per tree and the density of trees (number per hectare) over time (Matthews et al., 2016). Yield class 14 of *P. sitchensis* is chosen as species A since it is the dominant conifer species grown in the British uplands (Forestry Commission, 2011). The timber volume for species B is not fitted since parameter *R*_*P*_ allows the timber volume of species B (and/or the price of timber of species B) to be scaled relative to species A. This permits flexibility within the model since analysis of sensitivity to *R*_*P*_ can be explored.

### 3.2 Epidemiological system

If there is a disease outbreak then a SI compartmental model shows how the area occupied by infected trees of species A changes throughout time. The area occupied by species A, *L*_*A*_(*δ*) = (1 − *δ*)*L*, consists of the area of trees (of species A) that are susceptible to disease (i.e. not infected), *S*_*A*_(*t*, *δ*), and those that are infected, *I*_*A*_(*t*, *δ*), at time *t* (so *L*_*A*_(*δ*) = *S*_*A*_(*t*, *δ*) + *I*_*A*_(*t*, *δ*)). All trees of species A are initially susceptible to infection, giving *S*_*A*_(0, *δ*) = *L*_*A*_(*δ*). If a pathogen invades and there is a disease outbreak, then this occurs via some primary infection rate, *ϵ*, and once the pathogen has arrived in the plot, there is a secondary infection rate which represents the spread of infection throughout the forest. *ϵ* can be thought of as the external pressure of the pathogen/pest on the plot, for example, the rate of arrival of inoculum on the wind or insect vectors: an increase in *ϵ* increases the rate that susceptible trees in the plot will become infected. Note that there is no interaction between the probability of the pathogen arrival, *P*, and the primary infection rate, *ϵ* – we discuss this later.

To illustrate the pathogen transmission term we start with the assumption that the rate at which a single infected tree ‘converts’ a susceptible tree to an infected tree is

(11) $$g_{1}(L)\times g_{2}\left(\frac{L_{A}}{L}\right)\times g_{3}\left(\frac{S_{A}}{L_{A}}\right)$$

where *g*_1_(*L*) is the contact rate between the infected tree and any other tree in the plot, *g*_2_(*L*_*A*_/*L*) is the probability that the contact is with a tree of species A, and *g*_3_(*S*_*A*_/*L*_*A*_) is the probability that the contact is with a susceptible tree. (Note that whilst demonstrating how the pathogen transmission term is constructed, we simplify the notation of *L*_*A*_(*δ*) to *L*_*A*_, *S*_*A*_(*t*, *δ*) to *S*_*A*_ and *I*_*A*_(*t*, *δ*) to *I*_*A*_ for the sake of clarity.) The contact can be thought of as occurring through the spores spreading throughout the plot, or spread by the growth of the pathogen through the tree root network. Contact may occur at different spatial scales dependent on the dispersal range – but this will be proportional to the total area of the plot, *L* (not the area of species A). The probability that the contact is with a tree of species A will depend on how the tree species are arranged. For example, if the species are arranged as a monoculture, then the contact will always be with species A. However, if the two species are mixed intimately with no spatial correlations, then the probability of contact with a tree of species A will be proportional to the number (area) of trees of species A planted within the plot. This gives

(12) $$g_{2}\left(\frac{L_{A}}{L}\right)=\left\{\begin{array}{cc} 1 & \mathrm{if}\;a\;\mathrm{monoculture}\\ \frac{L_{A}}{L} & \mathrm{if}\;a\;\mathrm{mixture}. \end{array}\right)$$

Finally, the probability that contact is made with a susceptible tree is proportional to the number (area) of susceptible trees of species A (*S*_*A*_/*L*_*A*_).

Therefore, for a mixture of two species, the rate of ‘converting’ a susceptible area to an infected area using Eq. (11) is

(13) $$\left(g_{1}(L)\times \frac{L_{A}}{L}\times \frac{S_{A}}{L_{A}}\right)\times I_{A}=\beta S_{A}I_{A}$$

since *g*_2_(*L*_*A*_/*L*) = *L*_*A*_/*L* from Eq. (12) and *β* is the secondary infection rate. The rate of change of area of infected trees over time is therefore

(14) $$\frac{{\mathrm{dI}}_{A}}{\mathrm{dt}}=\beta \left(L_{A}(\delta )-I_{A}(t,\delta )\right)\left(I_{A}(t,\delta )+\epsilon \right)$$

since *S*_*A*_(*t*, *δ*) = *L*_*A*_(*δ*) − *I*_*A*_(*t*, *δ*). (This is the same as classical ‘density-dependent’ transmission (McCallum et al., 2001), where the force of infection increases with the area of infected trees – since the contact rate with species A is increased. Note also that we now change *L*_*A*_ to *L*_*A*_(*δ*), *S*_*A*_ to *S*_*A*_(*t*, *δ*) and *I*_*A*_ to *I*_*A*_(*t*, *δ*).) Using the initial conditions (*I*_*A*_(0, *δ*) = 0) Eq. (14) is solved to give

(15) $$I_{A}(t,\delta )=\frac{\epsilon \left(e^{(L_{A}(\delta )+\epsilon )\beta t}-1\right)}{(\epsilon /L_{A}(\delta ))e^{(L_{A}(\delta )+\epsilon )\beta t}+1},$$

where *L*_*A*_(*δ*) = (1 − *δ*)*L*.

When a monoculture of species A is planted (*δ* = 0 giving *L*_*A*_(0) = *L*), Eq. (15) simplifies to

(16) $$I_{A}(t,0)=\frac{\epsilon \left(e^{(L+\epsilon )\beta t}-1\right)}{(\epsilon /L)e^{(L+\epsilon )\beta t}+1}.$$

**Fig. 1:**
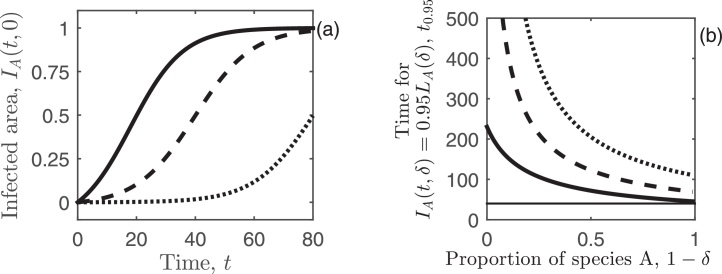
Area occupied by infected trees over time and the time taken for 95% of species A to become infected. In (a) the area of infected trees, *I*_*A*_(*t*, 0) (hectares), from Eq. (16) is shown over time, *t* (years), for a monoculture of species A (*δ* = 0 and *L*_*A*_(0) = *L*). In (b) the time taken for 95% of species A to become infected, *t*_0.95_ (years; with *θ* = 0.95 in Eq. (17)), is shown against the proportion of species A, 1 − *δ*, planted in the plot. The horizontal line indicates the rotation length, *T* = 40 years. In both panels the secondary infection rate is *β* = 0.1, and the primary infection rate is *ϵ* = 0.13 (solid), *ϵ* = 0.0175 (dashed) and *ϵ* = 0.00033 (dotted). Parameter values are given in Table 1.

Fig. 1(a) shows the area of infected trees over time for Eq. (16), for the three primary infection rates, *ϵ*, used in this study. If a monoculture of species A is planted, then as *t* → ∞, the infected area tends to the total area, *I*_*A*_(*t*, 0) → *L*. This can be shown by standard steady-state analysis for epidemic models, e.g. substituting *t* =∞ into Eq. (16).

Under the restrictions of planting a monoculture (Section 3.1.1), the pathogen transmission occurs across the whole plot of fixed size *L*_*A*_(0) = *L* (when species A is planted). However, when a mixture is considered (Section 3.1.2), the area of species A is changed to find the optimal planting proportion of both species. Varying the area of species A will clearly have an economic impact through an increased loss from planting a higher proportion of the area with species B, but also through reducing the speed that infection progresses through the population of species A due to the reduced probability of pathogen contact between infected trees of species A and conspecific trees (Eq. (12)). This can be seen by finding the time for a fraction *θ* of species A to become infected, which is

(17) $$t_{\theta }=\frac{1}{\beta (L_{A}(\delta )+\epsilon )}\ln\left(\frac{\theta L_{A}(\delta )+\epsilon }{\epsilon (1-\theta )}\right),$$

where *θ* ∈ [0, 1]. Fig. 1(b) shows the time taken for 95% of species A to become infected (*θ* = 0.95) against the proportion of species A planted (1 − *δ*) for three primary infection rates. Increasing the area of species A (increasing 1 − *δ*, or increasing the probability of pathogen contact between trees of species A, Eq. (12)), decreases the time taken for the disease to spread throughout the population of species A. For example, a pathogen which arrives early in the rotation will take approximately 44 years for 95% of 1 ha of species A (1 − *δ* = 1) to become infected, but the time taken to infect 95% of the occupied area is more than tripled when the area of species A is reduced to 0.1 ha (1 − *δ* = 0.1; the black curve representing *ϵ* = 0.13 in Fig. 1(b)). This interaction will clearly have important implications when finding the optimal planting strategy for two tree species.

Disease affects the timber benefit obtained from infected trees. The function ${\tilde{L}}_{A}(t,\delta )=S_{A}(t,\delta )+\rho I_{A}(t,\delta )$ (or ${\tilde{L}}_{A}(t,0)=S_{A}(t,0)+\rho I_{A}(t,0)$ for a monoculture of species A) captures the effective area of the forest occupied by species A effective area of trees of species A at time *t* in the presence of disease. The parameter *ρ* ∈ [0, 1] measures the effect of the disease on the timber value, so that when *ρ* = 0 the timber from diseased trees has no value, alternatively when *ρ* = 1 there is no difference in value between timber from infected and uninfected trees. This function is used in the objective function for planting a monoculture (Eq. (3)) and a mixture (Eq. (8)).

## 4 Results

In this section the optimal planting strategy is shown when the forest manager plants a monoculture in Section 4.1, and then a mixture in Section 4.2 when infected timber is worth nothing (*ρ* = 0). Analysis of sensitivity to *ρ* is undertaken in Section 4.3. Finally a summary of the effect that a difference in establishment cost (between species A and B) has on the optimal planting strategy is given in Section 4.4.

### 4.1 Monoculture

**Fig. 2:**
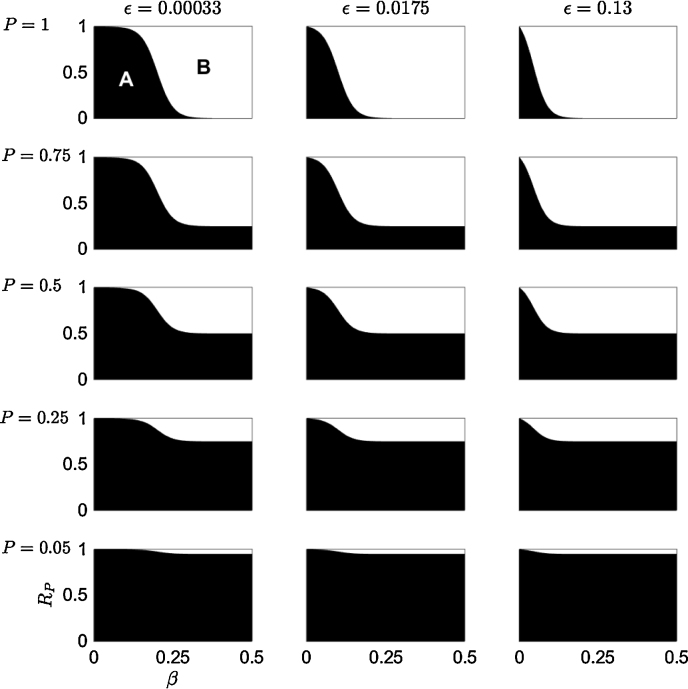
The optimal planting strategy under the conditions of planting a monoculture. The optimal planting strategy, s^*^, for a risk neutral manager is given by Eq. (5) and plotted in a *β* − *R*_*P*_ parameter space (the secondary infection rate *vs.* the timber value of species B relative to species A). The primary infection rate, *ϵ*, is altered between each column, and the probability of pathogen arrival, *P* is altered between each row. Only a monoculture can be planted: s^*^ = *A* (black region) or s^*^ = *B* (white region). Infected timber is worth nothing, *ρ* = 0, and all other parameter values are given in Table 1.

The optimal planting strategy for a monoculture, s^*^, is found by solving Eq. (5) when *ρ* = 0. The top row of Fig. 2 shows the variation in optimal planting strategy with the secondary infection rate (*β*; *x*-axis) and the timber value of species B relative to species A (*R*_*P*_; *y*-axis), for different primary infection rates (*ϵ*). As *β* is increased, there is an increase in the *R*_*P*_ range where s^*^ = *B*, since the loss from disease is increased, while the cost of planting species B remains the same. Once *β* reaches a level such that all trees in the plot become infected by the end of the rotation (*I*_*A*_(*T*, 0) = *L*), the optimal strategy will always be s^*^ = *B* independent of the difference in timber value. As the primary infection rate, *ϵ*, increases, the range of *R*_*P*_ where s^*^ = *B* increases for smaller values of *β* (shown by the boundary of the white parameter space moving towards the left with the increasing values of *ϵ* in Fig. 2). Again, this is due to the increase in the economic loss due to the disease.

When there is a lower probability of pathogen arrival, *P*, the expected loss from planting species A decreases. This is seen in Fig. 2 where the region in the parameter space where s^*^ = *A* increases as *P* decreases between the rows. Increasing *ϵ*, increases the parameter space where s^*^ = *B* across all of the values of *P* in Fig. 2. However, this effect diminishes with lower values of *P*, showing that it can still be optimal to have planted species A for large values of the secondary infection rate, *β*, especially for low values of *R*_*P*_. This occurs because the probability of economic loss due to the disease being realised is reduced with a lower probability of pathogen arrival.

An equation for the boundary between the two planting strategies is available by setting the objective function for both strategies to be equal (Eq. (3). This gives the relative timber value of species B, $R_{P}^{B}$, in terms of the primary and secondary infection rates. It can be expressed as

(18) $$R_{P}^{B}=1-P\frac{I_{A}(T,0)}{L},$$

since ${\tilde{L}}_{A}(T,0)=L-I_{A}(T,0)$ when *ρ* = 0. As the secondary infection rate, *β*, increases then $R_{P}^{B}\to 1-P$ since *I*_*A*_(*T*, 0) → *L*. This is shown in Fig. 2 as the *R*_*P*_ value of the boundary between the two species tends to 1 − *P* when *β* increases ($R_{P}=R_{P}^{B}$ at smaller values of *β* when *ϵ* is increased). Once the primary and/or the secondary infection rate is large enough for the infection to spread throughout the whole plot by the end of the rotation (*I*_*A*_(*T*, 0) = *L*), the optimal planting strategy is predominantly determined by the probability of arrival, *P*, and the timber value of species B relative to that of species A, *R*_*P*_.

### 4.2 Mixture

The optimal planting strategy for a mixture, δ^*^, is found solving Eq. (10). This can be found by differentiating Eq. (8) with respect to *δ*, which gives

(19) $$\frac{\mathrm{dE}[H(\delta )]}{d\delta }=-P\frac{d{\tilde{L}}_{A}}{d\delta }-L\left(P+R_{P}-1\right),$$

where

(20) $$\frac{d{\tilde{L}}_{A}}{d\delta }=-L-L\epsilon (\rho -1)e^{(L_{A}(\delta )+\epsilon )\beta T}\frac{\left(\epsilon /L_{A}{\left(\delta \right)}^{2})(e^{(L_{A}(\delta )+\epsilon )\beta T}-1)+\beta T(1+\epsilon /L_{A}(\delta )\right)}{{\left((\epsilon /L_{A}(\delta ))e^{(L_{A}(\delta )+\epsilon )\beta T}+1\right)}^{2}},$$

where *L*_*A*_(*δ*) = (1 − *δ*)*L*. Unfortunately, we cannot find δ^*^ explicitly from Eq. (19); however, we can proceed using numerical optimisation.

**Fig. 3:**
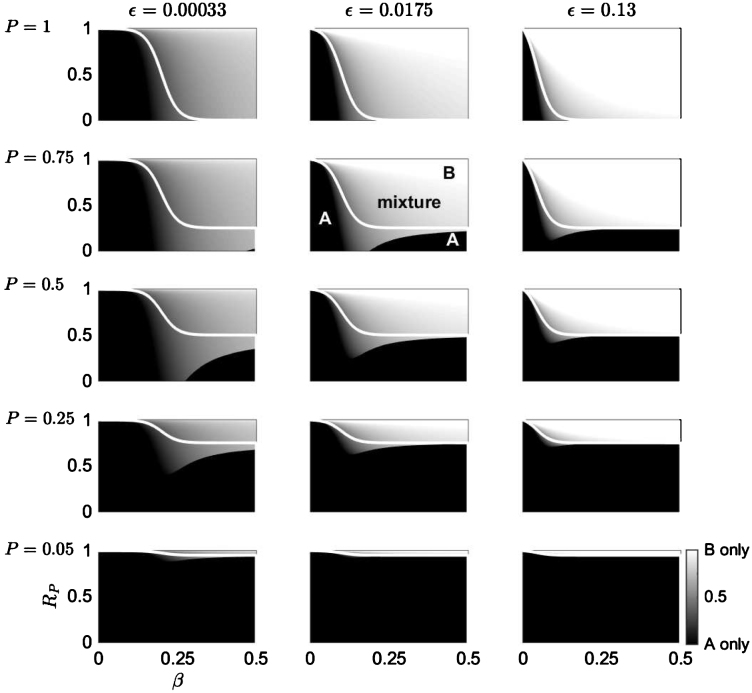
The optimal planting strategy under the conditions of a mixture. The optimal planting strategy, δ^*^, for a risk neutral manager is given by Eq. (10) and plotted in a *β* − *R*_*P*_ parameter space (the secondary infection rate *vs.* the timber value of species B relative to species A). The primary infection rate, *ϵ*, is altered between each column, and the probability of pathogen arrival, *P* is altered between each row. The grey scale (bottom right) shows δ^*^: a monoculture of species A when δ^*^ = 0 (black), of species B when δ^*^ = 1 (white) or a mixture of A and B when 0 < δ^*^ < 1 (gradations of grey). The white line indicates the switch in planting strategy when only a monoculture is allowed (i.e. the border between the black and white parameter spaces in Fig. 2). Infected timber is worth nothing, *ρ* = 0, and all other parameter values are given in Table 1.

The optimal planting strategy, δ^*^, is plotted in Fig. 3 against the secondary infection rate, *β*, and the timber value of species B relative to that of species A, *R*_*P*_, when infected timber is worth nothing *ρ* = 0. When the pathogen arrives late in the rotation (small primary infection rate, *ϵ*, left-hand column in Fig. 3), it will always be optimal to have planted a proportion of species A, and for a large region of the parameter space it is optimal to have planted a mixture. A lower probability of pathogen arrival increases the region of the parameter space where it is optimal to plant only species A (δ^*^ = 0, black). As the primary infection rate increases (center and right-hand columns of Fig. 3), the region in the parameter space where it is optimal to plant species A (either as a monoculture or in a mixture) decreases, and a region where it is optimal to plant a monoculture of species B emerges (δ^*^ = 1,white). Again, this occurs because the loss due to disease is increased as the primary infection rate (and/or the secondary infection rate) increases, whereas planting a higher proportion of species B reduces the overall loss.

It is interesting that when comparing Fig. 3 with the boundary between the two monoculture strategies (the white line given by Eq. (18) shown in Fig. 2), the region where it is optimal to plant a mixture (grey) extends into the parameter space where it was optimal to plant a monoculture of species B (to the right of $R_{P}^{B}$), much more than it extends into the parameter space where it was optimal to plant a monoculture of species A (to the left of $R_{P}^{B}$). Moreover, a region where it is optimal to plant a mixture for very small values of *R*_*P*_ and medium values of *β* emerges.

**Fig. 4:**
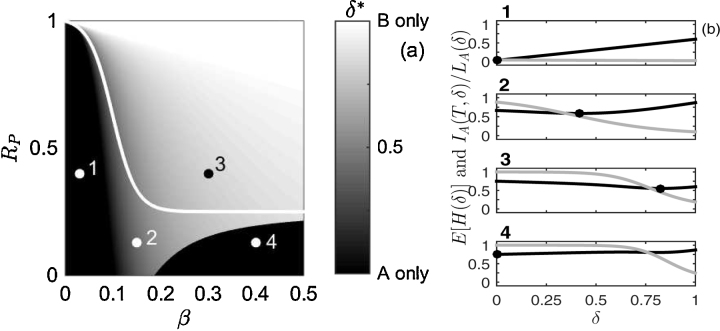
Sensitivity of the proportion of species A that is infected and the objective function to the proportion of species B planted. (a) The optimal planting strategy, δ^*^, given by Eq. (10) is plotted in a *β* − *R*_*P*_ parameter space (the secondary infection rate *vs.* the timber value of species B relative to species A) for a probability of pathogen arrival *P* = 0.75 and primary infection rate *ϵ* = 0.0175. The grey scale (on the right) shows δ^*^, and the white line indicates the switch in planting strategy, s^*^, when only a monoculture is allowed. In (b) the value of the objective function (*E*[*H*(*δ*)] in Eq. (8); black), and the proportion of species A that is infected (*I*_*A*_(*T*, *δ*)/*L*_*A*_(*δ*) from Eq. (15); grey) are shown against *δ* for the four selected regions in the parameter space in (a). The black dot on the *E*[*H*(*δ*)] curves in (b) indicates the minimum expected loss, and so gives the optimal value of *δ*. Parameter values are given in Table 1.

To explain this behaviour, the optimal planting strategy is explored in further detail for the case shown in Fig. 4. The value of the objective function (Eq. (8)) and the proportion of species A that is infected by the end of the rotation (Eq. (15)) are plotted against the proportion of species B planted, *δ*, in Fig. 4 (b) for the four points highlighted in Fig. 4 (a). At point 1, the secondary infection rate is small and only a small proportion of species A is infected by the end of the rotation (top plot in Fig. 4 (b)). The value of the objective function when a monoculture of species A is planted is therefore small (relative to the loss from planting any trees of species B) and a monoculture of species A is optimal, δ^*^ = 0. Decreasing the probability of pathogen arrival doesn’t affect the optimality of this solution since it will act to reduce the expected loss from disease (Fig. 3).

As the secondary infection rate increases, the expected loss due to disease from planting species A increases (and the loss from planting species B stays the same). It therefore becomes optimal to have planted a mixture of both species since, (i) timber from species B has a higher value than infected timber from species A (timber from infected trees is assumed to be worth nothing in this scenario), and (ii) planting species B reduces the secondary rate of infection to uninfected trees of species A. To expand on the second reason: as the area occupied by the trees of species A in the intimate mixture with species B decreases, the rate of contact decreases (Eq. (11) and Fig. 1 (b)), and so more uninfected – and higher value – trees of species A are available by the end of the rotation. The second plot in Fig. 4 (b) shows that increasing the proportion of species B in the mixture, *δ*, steadily decreases the proportion of infected trees of species A (grey), and initially decreases the value of the objective function (black) due to the higher proportion of trees of species B that remain uninfected due to the effects of mixing with species B. However, once *δ* = δ^*^ ≈ 0.4, the expected loss increases since the loss from planting more species B is greater than the benefit from reduced spread of infection to trees of species A. This provides the reason why it may be optimal to plant a mixture despite the very low values assumed in this scenario for timber from species B (in the region of the parameter space below the white $R_{P}^{B}$ boundary in Fig. 4 (a) where for a monoculture forest it would be optimal to plant only species A).

As the secondary infection rate is increased further, the spread of infection through the population of species A occurs faster. A region of the parameter space where it is optimal to plant a high proportion of species B emerges for high values of timber of species B, *R*_*P*_. The optimal planting proportion changes to a mixture as *R*_*P*_ is decreased (point 3), since planting a mixture reduces the spread of infection throughout species A (due to reduced probability of contact with other trees of species A, Eq. (12)), but only when the area occupied by trees of species A is very small (due to a higher *β* in this scenario). This can be seen for points 3 and 4 in Fig. 4 (b) where all the trees of species A are infected by the end of the rotation, which occurs when the proportion of the area occupied by trees of species A is approximately greater than 0.5 (*δ* < 0.5). When *δ* > 0.5, there is a reduction in *β* and thus in the proportion of the population of species A that is infected. Decreasing *R*_*P*_ further means that the loss from planting species B is now high, and thus there is a reduced benefit due to planting a mixture, and it therefore becomes optimal to plant a monoculture of species A, δ^*^ = 0, since there is a probability that the loss from disease may not be realised. This region is denoted by point 4 in Fig. 4 (a), and it can be seen that the upper boundary of this (black) region approaches the boundary $R_{P}^{B}=1-P$ of the region of the parameter space where, for a monoculture forest, it would be optimal to plant only species A (white curve).

### 4.3 Sensitivity to the revenue from the timber of infected trees relative to uninfected trees

**Fig. 5:**
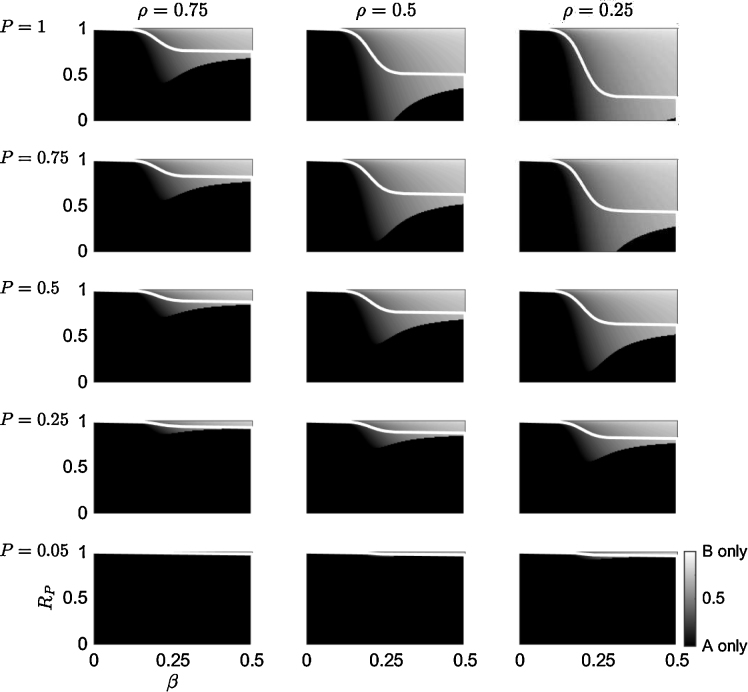
Sensitivity of the optimal planting strategy to changes in the revenue of timber from infected trees (relative to uninfected trees). The optimal planting strategy, δ^*^, for a risk neutral manager is given by Eq. ((10) and plotted in a *β* − *R*_*P*_ parameter space (the secondary infection rate *vs.* the timber value of species B relative to species A). The reduced value of timber from infected trees of species A, *ρ*, is altered between each column, and the probability of pathogen arrival, *P*, is altered between each row. The grey scale (bottom right) shows δ^*^: a monoculture of species A when δ^*^ = 0 (black), of species B when δ^*^ = 1 (white) or a mixture of A and B when 0 < δ^*^ < 1 (gradations of grey). The white line indicates the switch in planting strategy when only a monoculture is allowed. The primary infection rate is *ϵ* = 0.00033 and other parameter values are given in Table 1.

In this section the sensitivity of the optimal planting strategy to changes in the value of infected timber from species A, *ρ*, is qualitatively examined in Fig. 5 which shows the optimal planting strategy, δ^*^, when the values of *ρ* are varied between the columns, and values of the probability of pathogen arrival, *P*, are varied between the rows. When the pathogen is certain to arrive (top row in Fig. 5), decreasing *ρ* reduces the region in the parameter space where it is optimal to plant a monoculture of species A, δ^*^ = 0. This is not surprising since the loss from disease increases as *ρ* decreases, and therefore a greater proportion of species B is required to offset the loss (by reducing the spread of infection between trees of species A). As *P* is decreased, the region in the parameter space where δ^*^ = 0 increases (independently of the value of *ρ*).

When the primary infection rate, *ϵ*, is increased (results are not shown here), the optimal planting strategy's sensitivity to *ρ* remains qualitatively similar to the results shown in Fig. 5: decreasing *ρ* reduces the region in the parameter space where it is optimal to plant only species A. However, increasing *ϵ* decreases the region in the parameter space where it is optimal to plant a mixture, and a region emerges where it is optimal to plant a monoculture of species B (this is similar to the behaviour in Fig. 3 when *ϵ* is increased).

### 4.4 Difference in cost of establishment

We have also examined the scenario where the economic difference between planting the two species occurs at the beginning of the rotation through the cost of establishment instead of through the timber value at the end of the rotation. To do this we adjust the model to include the cost of establishment of both species (which is linearly dependent on the area of forest) and include a coefficient *R*_*C*_ which scales the cost of establishment of species B relative to species A (where *R*_*C*_ ≥ 1 since we assume that the disease-resistant species B is more expensive to establish than the susceptible species A). The timber benefit from species B is now the same as the timber benefit from uninfected timber of species A, and the timber benefit from species A will be reduced due to disease. We found that the key bioeconomic parameters had a similar effect on the optimal planting strategy when compared with the model in which the economic difference between planting the two species occured through the timber benefit (Section 4.2). More specifically, when the rate of secondary infection, *β*, is small, the loss from disease is at a minimum and so it will be optimal to plant only species A. As *β* increases, the loss from disease increases; however, planting a mixture will reduce the probability of contact with other trees of species A, and so it will become optimal to plant a mixture for a range of values of *R*_*C*_. Increasing *β* further means that the infection will spread throughout the area of species A by the end of the rotation, and so the range of *R*_*C*_ values where is it optimal to plant a mixture reduces. Further, decreasing the probability of pathogen arrival increases the range of *R*_*C*_ values where it is optimal to plant species A.

## 5 Discussion

Our bioeconomic model shows that diversifying tree species composition can reduce the expected negative economic impacts of a pathogen on a forest, and that this effect is dependent on the pathogen's characteristics (probability of arrival, time of arrival, and rate of spread of infection) and the losses (damage of the disease to the susceptible species and reduced benefit due to planting the resistant species). Indeed, reduction of damage by a pathogen is just one reason why tree species diversification in forests is currently being advocated. Other benefits include, but are not limited to: improved overall biomass through mixture overyielding (Smith et al., 2013, Piotto, 2008, Kelty, 2006); improved market resilience using, for example, portfolio theory (Neuner et al., 2013, Roessiger et al., 2013, Knoke and Seifert, 2008); decreased wind throw or storm damage (Felton et al., 2016, Jactel et al., 2009, Schütz et al., 2006, Knoke et al., 2005); aiding adaptation to a changing climate (Felton et al., 2016, Cameron, 2015, Pawson et al., 2013); and reducing pest population sizes and damage (Griess and Knoke, 2011, Jactel et al., 2005, Jactel and Brockerhoff, 2007). However, it is often difficult to generalise about the benefits of diversification as individual studies concentrate on different specific systems. This is in part recognised by the number of papers with conflicting findings, which suggests that there may not always be benefits to planting mixtures and, in some circumstances, there may be negative effects. For example, Griess and Knoke (2011) discussed that tree species with similar ecological niches will not produce a greater yield when planted together since they may be competing for similar resources (Chen et al. (2003) also suggests the effect of tree mixing on yield is very site and species dependent). Felton et al. (2016) highlighted that fire risk could actually increase with some mixtures if, for example, shade levels were altered such that understorey vegetation is promoted (which can act as a fuel). Moreover, whilst species mixtures have been shown to reduce pest outbreaks though mechanisms such as associational resistance (Jactel et al., 2009, Tahvanainen and Root, 1972), there are many other studies which argue that increasing the number of tree species may facilitate invasion from more generalist herbivores (Plath et al., 2012, Koricheva et al., 2006). Often outcomes are dependent on the mixture selected and the productivity of the site (Felton et al., 2016); this highlights the importance of both primary empirical studies and meta-analyses of their results when trying to understand the effect of diversification on the forest system.

Bioeconomic models like the one presented here can also add to this important discussion because they (1) provide a broader perspective on how different biological and economic characteristics qualitatively affect the optimal planting strategy, and (2) provide a flexible (and extendable) framework so that the optimal planting strategy for specific host–pathogen systems can be examined (often using data from empirical studies). In the present study's models we assumed that only one of the two tree species was susceptible to a pathogen. In reality, and due to the timescale of the rotation, both species may be susceptible to different pathogens or indeed to the same pathogen if there is some evolutionary change in the invader (such as for *P. ramorum*; Appiah et al., 2004). Our model can be extended to include this by altering the epidemiological system and the objective function appropriately. However, we suggest that much caution is needed since it is not clear what effect the two tree species being susceptible to different pathogens would have on the pathogen transmission for each species (and possibly between species if the same pathogen can infect both). This complexity is highlighted in the following discussion regarding the pathogen transmission term used in this study.

The effect of how to characterise the pathogen transmission term within compartmental models has been widely debated within epidemiological modelling (e.g. Begon et al., 2002, McCallum et al., 2001). In this instance, our model uses a pathogen transmission term which is derived using the contact rate, the probability of contact with tree of a species that is susceptible to the pathogen, and the probability that the tree which belongs to the susceptible species is susceptible to the pathogen (i.e. not already infected). In our study, it is assumed that the probability of spores contacting a susceptible tree is proportional to the fraction of the trees in the forest which are susceptible to a pathogen (i.e. *L*_*A*_(*δ*)/*L*). Whilst we do not specify the specific spatial arrangement of the tree species in the forest within this paper, the ‘density-dependent’ transmission term used can satisfactorily represent a forest where trees are intimately mixed. Changing the spatial arrangement of the forest significantly may affect the probability of a pathogen spore contacting a tree of a conspecific species, and thus require alteration of the pathogen transmission term. For example, if the two species are each planted in a separate block, then the probability of contact between conspecific trees may be higher; in fact it may even be one. In turn this would alter the pathogen transmission term since *g*_2_(*L*_*A*_(*δ*)/*L*) = 1 (Eq. (12)), and the commonly used ‘frequency-dependent’ transmission term is derived, giving

(21) $$\frac{{\mathrm{dI}}_{A}}{\mathrm{dt}}=\beta \left(L_{A}(\delta )-I_{A}(t,\delta )\right)\left(\frac{I_{A}(t,\delta )}{L_{A}(\delta )}+\epsilon \right).$$

Analysis of our bioeconomic model using Eq. (21) shows that it would never be economically optimal to plant a mixture (since no reduction in the probability of contact, and thus spread of infection, is gained by planting two species in separate blocks).

This highlights three important points. Firstly, more evidence of how tree pathogens spread within multi-species forests is crucial for understanding how this affects management strategies like tree species composition. Secondly, careful construction and interpretation of bioeconomic models is essential. Finally, how do different spatial arrangements within a forest change the optimal planting strategy in the presence of a tree pathogen? This final point would require further study using a bioeconomic model at the individual tree-level in order to incorporate the detail required. Moreover, if the spatial structure was representative of arrangements used in practice, then costs (and benefits) of species composition could be included in more detail. We ignore the potential for increased costs of planting a mixture (through, for example, a difference in combined timber yield or an increased cost of extraction), since this adds an unnecessary complexity, and is likely to depend on the species and their arrangement within the forest. However, extending the model framework we present here to be spatially-explicit would allow this detail to be examined and would be an important contribution to the literature, with direct relevance to forestry practice.

At the other end of the spatial scale, if mixtures reduce the spread of infection within a forest, then this may also reduce the spread between forests since there is less infection pressure being emitted by, say, spores. This is an important question at the landscape scale since reduction in the spread between forests could ‘buy time’, which may reduce the overall damage by allowing trees to grow more before being infected, and so increase the economic benefit of salvageable timber. The effect of mixtures has important policy implications since advice, incentive mechanisms or even regulations could be altered in favour of tree species mixtures, not only to reduce damage within individual forests, but also to reduce the spread of infection at a landscape and even regional level. This study only addresses effects of a pathogen on timber benefits within a single forest. To understand the effect of diversifying at a landscape scale, a bioeconomic model could be used to examine how different species mixtures affect the spread of infection on a network of connected forests. This could be analysed from either the perspective of an individual forest manager (who manages a single forest in the network by minimising their expected loss, as in this study), or from a social planner perspective (where the objective is to minimise the expected loss across all forests at a landscape level).

In the model presented here there is no interaction between the probability of the pathogen arrival and the time at which the pathogen arrives. This has two important benefits. Firstly, it allows separate sensitivity analyses of how each of these parameters affects the optimal planting strategy. Secondly, it is likely that a forest manager may separate the probability of the pathogen arriving and the time of arrival when making decisions regarding the threat of a specific pathogen. As some diseases, such as *H. annosum*, have a relatively low probability of spreading between isolated forests, whereas it is considered that others, such as *Hymenoscyphus fraxineus*, will innevitably spread to all forests containing the host tree *Fraxinius exclesior* in the UK, but the greater uncertainty is when this will occur to an inidividual forest. We note, however, that the deterministic model framework excludes any uncertainty in the time of arrival and spread of the pathogen, which would be seen in the field. A common way of including this uncertainty is to use a stochastic process, such as a Poisson process. We have carried out simulations using a Poisson process to introduce the pathogen to the forest, and obtained very similar results to those obtained with the main method described in this paper. Incorporating full stochasticity into the model would be an interesting extension; however, it is beyond the scope of this paper.

A further consideration is non-timber benefits produced by forests. In this study we have excluded these by concentrating on plantations managed for the dominant purpose of timber production. However, it is acknowledged that there are a range of non-timber outputs associated with plantation forestry, such as carbon sequestration, water regulation and habitat provision (Bauhus et al., 2011). Diversification of tree species composition is commonly linked to increasing the range of ecosystem services provided (Gamfeldt et al., 2013); however, tree pathogens can often have an adverse effect on these (Pimentel et al., 2005). Quantifying the non-timber benefits, and the effect that the interaction of tree species’ diversification and tree pathogens have on them, is likely to be difficult. However, bioeconomic models could be used to explore a range of effects on timber and non-timber benefits, and how these change the optimal planting strategy. Analysis of sensitivity to the level of non-timber benefits would provide a useful comparison of how the optimal strategy for a plantation forest managed only for timber benefits compares with a multi-output forest. One way of examining this could be to extend the objective function presented here to include a non-timber term that is dependent on the number of species planted and also the effect of the disease on the non-timber benefit. (Another possibility is for the non-timber benefits to be linked to the social planner model mentioned above, since the provision of non-timber benefits is often dependent on the connectivity of forests, for example habitat for wildlife corridors (Lookingbill et al., 2010, Dobson et al., 1999).)

## 6 Conclusions

We develop a novel approach using a bioeconomic model to assess the effect of tree disease on optimal planting strategy for tree species mixtures. To find the optimal planting proportion of two trees species – of which one is resistant to disease and the other susceptible – we minimise the reduction in timber benefit due to disease by increasing the proportion of a second tree species that has a lower timber benefit compared with the susceptible tree species (in the absence of infection).

A key result of this paper is that we found that the risk and damage of disease can alter the optimal planting proportion. If the forest manager perceives that the risk of a pathogen arriving is zero, they will only plant the species which has the highest net benefit (due either to its higher timber benefit or lower establishment costs). If the forest manager wishes to plant such a single-species monoculture where there is a risk of the pathogen arriving, then the rate of primary and secondary infection increases the probability that the manager should plant only the resistant tree species, despite its lower net benefit, in place of the susceptible species (Fig. 2). The probability of pathogen arrival also affects which species it is optimal to plant: as the probability decreases, the benefit of planting the susceptible species is greater since the expected damage due to disease is reduced (Fig. 2).

When the forest manager has the option of planting a mixture of both tree species, the optimal planting proportion is dependent on the probability of pathogen arrival, the rate of primary and secondary infection, the effect of disease on the timber value, and the reduced benefit of planting the resistant species (relative to the susceptible tree species, in the absence of infection). For a pathogen that has a small rate of primary and/or secondary infection, the optimal planting strategy is to plant a monoculture of the susceptible species since the damage caused by the disease is small (Fig. 3). As the rate of secondary infection increases, it becomes optimal to plant a mixture of both species, predominately because introducing the resistant species will reduce the probability that the pathogen will infect a tree which is susceptible to disease (Eq. (10). This is akin to the ‘dilution effect’ where the ability of a pathogen establishing and transmitting between susceptible hosts is reduced by species diversity (Keesing et al., 2006). This is a key result: planting a tree species mixture will increase the overall net benefit even if the benefit from the disease resistance of the second species is small (Fig. 3, Fig. 4). Increasing the secondary infection rate will again reduce the benefit of planting a mixture and it will be optimal to plant only the resistant species. However, a decrease in the probability of the pathogen's arrival will reduce the expected loss due to the disease, and so it may be optimal to plant only the susceptible species (dependent on the difference in benefit between the resistant species and uninfected trees of the susceptible species; Fig. 3). Reducing the effect of disease on the timber benefit of the susceptible species will increase the proportion of the susceptible species that it is optimal to plant (Fig. 5).

In the final part of this study we examined the case where the difference between uninfected trees of a susceptible species and a resistant tree species occurs at the beginning or end of the rotation (through a difference in establishment costs or timber benefit respectively). We found that the sensitivity of the optimal planting strategy to the different pathogen characteristics behaved similarly. This showed that the qualitative changes in the optimal planting strategy are independent of whether the difference between the two species occurs at the beginning or end of the rotation; however, we have not examined the effect of this difference on the value of the net benefit of the optimal solution. One extension to this model would be to examine the case where the resistant species is more expensive to establish *and* has a reduced timber value (compared with the uninfected, susceptible species). Moreover, it is interesting to note that when the difference between the uninfected, susceptible tree species and resistant tree species occurs at the end, the optimal planting strategy is not dependent on the discount rate. However, when the difference occurs at the beginning (due to the difference in establishment costs), then the optimal planting strategy may be dependent on the discount rate. Increasing the discount rate will decrease the expected timber benefit (from both species), but the effect on the optimal planting strategy is not clear, thus sensitivity of the results to the discount factor should a future research priority.

Most previous modelling/statistical work on this topic is for specific host–pathogen systems and uses data from the field (Guyot et al., 2016, Hantsch et al., 2014, Haas et al., 2011, Thor et al., 2005, Gerlach et al., 1997). Therefore, this paper makes a step-change advance on existing capacity to assess the effect of diversification of production forests with respect of emerging pathogens through a general framework to analyse the impact of economic and biological conditions on the optimal planting strategy in the presence of tree disease. This flexible model framework can be parameterised (and extended) to represent a specific host–pathogen system, which would allow the optimal planting strategy to be examined for threats of new pathogens.

## Figures and Tables

**Table 1:** The parameter definitions and baseline values used in this paper.

Parameter	Definition	Baseline value/range
ECONOMIC		
*p*	Price of timber from species A (£ m^−3^)	*p* = 18.24^a^
*R*_*p*_	Price of timber of species B (relative to A)	*R*_*p*_ ∈ [0, 1]
*R*_*c*_	Cost of establishment of species B (relative to A)	*R*_*c*_ ∈ [1, 3]
*r*	Discount rate	*r* = 0.03

ECOLOGICAL		
*T*	Fixed rotation length (years)	*T* = 40
*t*	Time (years)	*t* ∈ [0, *T*]
*L*	Total area of forest (ha)	*L* = 1
*δ*	Planting proportion of species B	*δ* ∈ [0, 1]
*L*_*A*_(*δ*)	Area of trees from species A (ha)	*L*_*A*_(*δ*) = (1 − *δ*)*L*
*L*_*B*_(*δ*)	Area of trees from species B (ha)	*L*_*B*_(*δ*) = *δL*
*f*(*T*)	Timber volume per unit area (m^3^ ha^−1^)	*f*(*T*) = 579.9

EPIDEMIOLOGICAL		
*P*	Probability of pathogen arrival	*P* ∈ [0, 1]
*ϵ*	Primary infection rate (ha)	*ϵ* = {0.00033, 0.0175, 0.13}
*β*	Secondary infection rate (ha^−1^ yr^−1^)	*β* = 0.1
*ρ*	Reduction in timber value of infected trees	*ρ* = 0
	relative to uninfected trees	
*I*_*A*_(*T*, *δ*)	The area of infected forest of species A (ha)	Eq. (14)
*S*_*A*_(*T*, *δ*)	The area of susecptible forest of species A (ha)	*S*_*A*_(*T*, *δ*) = *L*_*A*_(*δ*) − *I*_*A*_(*T*, *δ*)
${\tilde{L}}_{A}(T,\delta )$	Effective area of the forest occupied by species A when disease is present (ha)	${\tilde{L}}_{A}(T,\delta )=S_{A}(T,\delta )+\rho I_{A}(T,\delta )$
*θ*	Fraction of species A to become infected	*θ* ∈ [0, 1]
*t*_*θ*_	Time for *θ* of species A to become infected (years)	Eq. (17)

a The price of timber is the average standing price (per cubic metre overbark) taken from Coniferous Standing Sales Price Index on 30th September 2015 for Great Britain (http://www.forestry.gov.uk/forestry/INFD-7M2DJR).
